# Integration of an interpretable machine learning algorithm to identify early life risk factors of childhood obesity among preterm infants: a prospective birth cohort

**DOI:** 10.1186/s12916-020-01642-6

**Published:** 2020-07-10

**Authors:** Yuanqing Fu, Wanglong Gou, Wensheng Hu, Yingying Mao, Yunyi Tian, Xinxiu Liang, Yuhong Guan, Tao Huang, Kelei Li, Xiaofei Guo, Huijuan Liu, Duo Li, Ju-Sheng Zheng

**Affiliations:** 1 Institute of Basic Medical Sciences, Westlake Institute for Advanced Study, School of Life Sciences, Westlake University, 18 Shilongshan Rd, Cloud Town, Hangzhou, 310024 China; 2 China-Canada Joint Lab of Food Nutrition and Health, Beijing Technology and Business University, Beijing, China; 3 Department of Obstetrics and Gynecology, Hangzhou Women’s Hospital (Hangzhou Maternity and Child Health Care Hospital), Hangzhou, China; 4 Department of Epidemiology and Biostatistics, School of Public Health, Zhejiang Chinese Medical University, Hangzhou, China; 5 Jiaxing University Affiliated Women and Children Hospital, Jiaxing, China; 6 Department of Epidemiology and Biostatistics, School of Public Health, Peking University, Beijing, China; 7 Institute of Nutrition and Health, Qingdao University, 308 Ningxia Road, Qingdao, China

**Keywords:** Preterm infants, Childhood obesity, Early life risk factors, Machine learning

## Abstract

**Background:**

The early life risk factors of childhood obesity among preterm infants are unclear and little is known about the influence of the feeding practices. We aimed to identify early life risk factors for childhood overweight/obesity among preterm infants and to determine feeding practices that could modify the identified risk factors.

**Methods:**

A total of 338,413 mother-child pairs were enrolled in the Jiaxing Birth Cohort (1999 to 2013), and 2125 eligible singleton preterm born children were included for analyses. We obtained data on health examination, anthropometric measurement, lifestyle, and dietary habits of each participant at their visits to clinics. An interpretable machine learning-based analytic framework was used to identify early life predictors for childhood overweight/obesity, and Poisson regression was used to examine the associations between feeding practices and the identified leading predictor.

**Results:**

Of the eligible 2125 preterm infants (863 [40.6%] girls), 274 (12.9%) developed overweight/obesity at age 4–7 years. We summarized early life variables into 25 features and identified two most important features as predictors for childhood overweight/obesity: trajectory of infant BMI (body mass index) *Z*-score change during the first year of corrected age and maternal BMI at enrollment. According to the impacts of different BMI *Z*-score trajectories on the outcome, we classified this feature into the favored and unfavored trajectories. Compared with early introduction of solid foods (≤ 3 months of corrected age), introducing solid foods after 6 months of corrected age was significantly associated with 11% lower risk (risk ratio, 0.89; 95% CI, 0.82 to 0.97) of being in the unfavored trajectory.

**Conclusions:**

The trajectory of BMI *Z*-score change within the first year of life is the most important predictor for childhood overweight/obesity among preterm infants. Introducing solid foods after 6 months of corrected age is a recommended feeding practice for mitigating the risk of being in the unfavored trajectory.

## Background

Over the past decades, about 1 in 10 of the babies were born preterm (defined as delivery at < 37 completed weeks of gestation) every year globally and more than 80% of the preterm births occurred in Asia and sub-Saharan Africa [1]. China was one of the top 5 countries for estimated number of preterm births and accounted for 7.8% of preterm births globally in 2014 [1]. As the quality of care for preterm infants improves and preterm survival rates increase [2], maintenance of a healthy metabolic status for the preterm infants over time has become a common research interest.

Preterm infants are at a higher risk of developing childhood obesity compared with term infants [3]. However, risk factors of childhood obesity among this specific population of infants are still unclear [4–7]. Prospective birth cohort study with a large sample size and a long follow-up period are undoubtedly ideal for addressing the question. However, the abundant, complex, high-dimensional, and heterogeneous health care data (e.g., biomedical and lifestyle data) pose a challenge to traditional data processing, statistical analysis based on a priori assumption, and result interpretation. Machine learning can help reveal relationships from the data without the need to define them a priori and derive predictive models without a need for strong assumptions about the underlying mechanisms [8, 9]. Furthermore, understanding why a predictive model made a specific prediction or explaining the specific features that lead to the prediction is even more clinically meaningful as some factors may be modifiable.

In the present study, we used an interpretable machine learning tool to identify early life risk factors of future overweight/obesity among singleton prematurely born children based on data collected over 14 years in a Chinese prospective birth cohort. As a secondary objective, we explored the associations between children’s feeding practices and the identified risk factors.

## Methods

### Study population

The Jiaxing Birth Cohort is a prospective cohort involving 338,413 mother-child pairs from Jiaxing, Zhejiang province (a middle-income area in southeast China), who were enrolled between 1999 and 2013. The enrolled women were followed up via visiting clinics until the birth of their children, and the children were continued to be followed up at ages 3, 6, 9, and 12 months during infancy stage, every 6 months between ages 12 and 36 months during toddler stage, and thereafter every year before they went to school (6–7 years of age) [10].

**Fig. 1:**
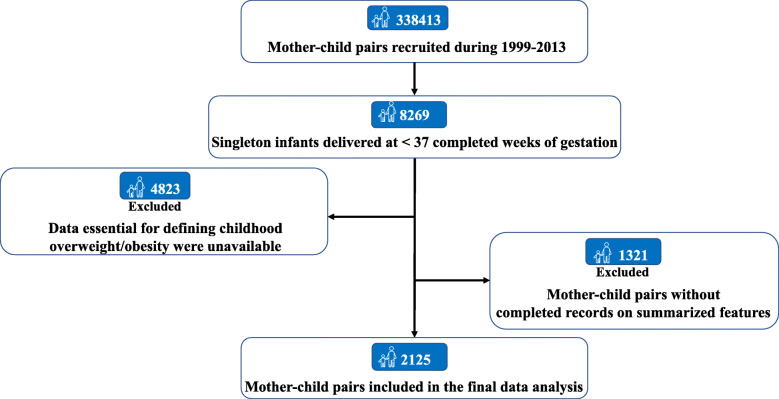
Flowchart of selection process of eligible participants from the Jiaxing Birth Cohort

A total of 8269 singleton children who were born before 37 completed weeks of gestation were screened from all 338,413 children in the Jiaxing Birth Cohort. We then retrieved classical items of anthropometric parameters, lifestyle factors and medical history, and excluded the mother-child pairs’ missing complete data to define childhood (at age 4–7 years) overweight/obesity (*n* = 4823) and those who lacked the data on any extracted item (*n* = 1321). Thus, the dataset from the remaining 2125 mother-child pairs were included in the present analyses (Fig. 1).

### Measurement of pre- and postnatal antecedents and ascertainment of overweight and obesity

Maternal demographic characteristics (e.g., age, education, occupation), maternal anthropometrics (e.g., body weight, height, blood pressure), perinatal clinical history (e.g., delivery mode, gestational age, birth weight, birth length), laboratory tests (e.g., hemoglobin concentration), postnatal feeding practices (e.g., duration of breast-feeding, use of formula, and timing of introducing solid foods), and growth patterns were recorded at their visits to local clinics.

For children at corrected ages between 4 and 5 years, *Z*-scores of body mass index (BMI)-for-age were calculated according to the 2006 WHO Child Growth Standards, and overweight and obesity were defined as the BMI *Z*-score between 2 and 3 and > 3, respectively [11, 12]. The 2007 WHO Child Growth standards were used to calculate *Z*-scores of BMI-for-age for children older than 5 years (corrected age), and overweight and obesity were defined as the BMI *Z*-score between 1 and 2 and > 2, respectively [12, 13].

### Data integration and predictor implementation

As the collected early healthcare data may have a variety of complex nonlinear interactions, we used a model based on a gradient boosting framework—LightGBM—to link input features with future overweight or obesity. LightGBM was developed to improve the efficiency and scalability of the gradient boosting machines (GBM) [14]. By adopting two novel techniques, Gradient-based One-Side Sampling (GOSS) and Exclusive Feature Bundling (EFB), LightGBM had a faster training speed, better accuracy, and higher efficiency compared to traditional gradient-boosting machines. With GOSS, a significant proportion of data instances with small gradients were excluded, and only the rest were used to estimate the information gain. GOSS was verified obtaining quite accurate estimation of the information gain with a much smaller data size. EFB was used to bundle mutually exclusive features, which was proven to reduce the number of features without hurting the accuracy of split determination by much [14].

### Model interpretation

Machine learning method usually only informs the results without telling us how it makes a certain decision. To solve this problem, we used a novel unified framework, SHAP (Shapley Additive exPlanations), to interpret predictions [15]. The impact of each feature on the model is represented using Shapley values, which are from coalitional game theory and consider all possible predictions for an instance using all possible combinations of inputs, and the average contribution of a feature value to the prediction is calculated in different coalitions [15].

### Statistical analysis

We used a latent-class growth model to track the changes of infant BMI *Z*-score during the first year of corrected age and cluster the pattern of changes into three distinct trajectories [16, 17]. Similarly, the maternal BMI changes, blood pressure changes, and hemoglobin concentration changes during pregnancy were all clustered into three distinct trajectories using the same method. Collectively, the retrieved items, including infant and maternal variables and clustered trajectories, were summarized into 25 features (Supplemental Table 1), which were subsequently used to construct the machine learning prediction model.

To ensure stability and extrapolation of the machine learning model, we randomly divided the dataset into separate training (*n* = 1143), validation (*n* = 381), and test sets (*n* = 382) at a ratio of 6:2:2. After fitting the parameters of the LightGBM model using the training set, we then validated and tuned the model among the validation set and evaluated the final performance using the independent test set. Receiver operating characteristic (ROC) curves were derived based on the validation and testing set, and the area under the curve (AUC) with 95% CI was calculated to evaluate the performance of the model.

We used the Tree SHAP implementation integrated into LightGBM to interpret the entire dataset. Features were sorted by the mean absolute SHAP values, across all samples. We selected features with an average absolute SHAP value greater than 0 as predictor variables. DeLong’s test for correlated ROC curves was used to assess the differences between models including all features and selected features only [18]. R package pROC was used for ROC curve analyses [19]. Then, we examined the marginal effect of each selected feature on prediction outcome after accounting for the average effect of all other features, as to investigate how the changes in a single selected feature affected the output of the machine learning model. As the SHAP value represents a feature’s responsibility for a change in the model output, we created a SHAP dependence plot to show the effect of a single feature across the whole dataset.

In order to examine the influence of overweight/obesity definition on the performance of our model, we performed sensitivity analyses by repeating our analyses after redefining the childhood overweight/obesity according to criteria which was used to screen overweight and obesity in Chinese children [20]. To further explore the potential feature selection bias, we also repeated the feature selection analysis using an ensemble feature selection tool (EFS), which made use of multiple feature selection methods and combined their normalized outputs to quantitative ensemble importance [21].

We then examined whether the machine learning-identified early life risk factor (i.e., trajectories of BMI *Z*-score) could be used as a preventive target of childhood overweight/obesity by improving infant feeding practices (e.g., duration of breast-feeding and timing of introducing solid foods). To this end, we examined the association between modifiable feeding practices and trajectories of BMI *Z*- score change, adjusted for mode of delivery, age at birth of offspring, gestational age, maternal education status, occupation, parity, maternal BMI at enrollment, maternal smoking status, maternal drinking status, and newborn birth weight. RR (95% CI) of unfavored BMI *Z*-score trajectories (defined as the trajectories that have a positive SHAP value, which corresponds to a higher risk of childhood overweight/obesity than those with a negative SHAP value) with the feeding practices (treated as categorical variables) were assessed by using a Poisson regression model.

We performed a mediation analysis to examine whether the trajectories of BMI *Z*- score change mediated the association between timing of introducing solid foods and childhood BMI *Z*-scores. We tested the associations of the trajectories of BMI *Z*- score change with the timing of introducing solid foods and childhood BMI *Z*-scores, using a linear regression model. A *sgmediation* command in STATA was used to calculate total, direct, and indirect effects, and the Sobel test was used to test the significance of indirect effect [22]. We used bootstrapping with 1000 sampling replications to estimate the 95% CI and calculate the proportion of the total effect of the timing of introducing solid foods on the childhood BMI *Z*-scores that was mediated by the trajectories of BMI *Z*-score change. The mediation models were adjusted for mode of delivery, gestational age, age at birth of offspring, maternal education status, occupation, parity, maternal BMI at enrollment, maternal smoking status, maternal drinking status, and newborn birth weight. Statistical analyses were done in STATA (version 15, Stata Corp, College Station, TX, USA), and a two-tailed *p* value < 0.05 was considered statistically significant.

## Results

### Population characteristics

A total of 2125 preterm infants with a median gestational age of 36 weeks (IQR 35–36) and a median follow-up of 6.4 years (IQR 5.8 to 6.8) were included in the final analyses. Two hundred seventy-four (12.9%) preterm infants developed overweight/obesity at 4–7 years old, and the number of cases increased from 13 at 4–5 years to 156 at 6–7 years (Supplemental Figure 1). Mothers of the children who progressed to childhood overweight/obesity had a younger age of menarche, higher BMI at enrollment, and distinct pattern of BMI changes compared with their counterparts (Table 1). Overweight/obese children were more likely to be boys, were delivered by cesarean section, and had heavier birth weight and distinct trajectory of BMI *Z*-scores during the first year of corrected age compared with those with normal weight (Table 1). Compared to excluded mother-child pairs, mothers included in the analysis were less likely multiparous, and the characteristics were generally balanced between the two datasets (Supplemental Table 2).

**Table 1:** Characteristics of preterm infants and their mothers by future childhood adiposity status at age 4 to 7 years

Characteristics	Offspring without overweight/obesity (***n*** = 1851)	Offspring with overweight/obesity (***n*** = 274)	***p***^§^
Boys	1076 (58.1)	186 (67.9)	< 0.01
Median (IQR) gestational weeks	36 (35–36)	36 (35–36)	0.89
Mean (SD) fetal heart rate at the last assessment	140.9 (6.3)	141.4 (5.7)	0.17
Cesarean delivery	1087 (58.7)	181 (66.1)	0.02
Mean (SD) birth weight	2.7 (0.5)	2.9 (0.5)	< 0.01
Mean (SD) birth length	48.2 (2.4)	48.6 (2.5)	0.02
Mean (SD) Apgar score at 1 min	8.5 (1.5)	8.6 (1.6)	0.37
Mean (SD) Apgar score at 5 min	9.1(1.1)	9.3 (1.0)	0.08
Duration of breast-feeding			0.06
< 1 month	735 (39.7)	94 (34.3)	
1–3 months	397 (21.4)	78 (28.5)	
4–5 months	678 (36.6)	95 (34.7)	
> 6 months	41 (2.2)	7 (2.6)	
Formula-feeding (ever)	1773 (95.8)	254 (92.7)	0.02
Timing of solid foods introduction			0.09
≤ 3 months	1235 (66.7)	180 (65.7)	
4–6 months	554 (29.9)	91 (33.2)	
> 6 months	62 (3.3)	3 (1.1)	
Trajectory of BMI *Z*-score change during the first year of corrected age			< 0.01
Trajectory 1	691 (37.3)	6 (2.2)	
Trajectory 2	1131 (60.1)	38 (13.9)	
Trajectory 3	47 (2.5)	230 (83.9)	
Mean (SD) maternal age at pregnancy	25.3 (4.2)	24.9 (4.0)	0.12
Mean (SD) maternal age of menarche	14.7 (1.3)	14.5 (1.3)	< 0.01
Parity (multiparous)	357 (19.3)	40 (14.6)	0.06
Maternal education			0.46
< High school	1357 (73.3)	191 (69.7)	
High school	325 (17.6)	55 (20.1)	
> High school	169 (9.1)	28 (10.2)	
Maternal occupation			0.08
Farm work/housework	1225 (66.2)	170 (62.0)	
Routine job	328 (17.7)	44 (16.1)	
Temporary work	138 (7.5)	24 (8.8)	
Others	160 (8.6)	36 (13.1)	
Median (IQR) gestational age at enrollment	10.0 (8.3–12.3)	10.1 (8.1–12.1)	0.59
Mean (SD) maternal BMI at enrollment	20.9 (2.8)	22.0 (3.2)	< 0.01
Trajectory of BMI change during pregnancy			< 0.01
Trajectory 1	686 (37.1)	70 (25.5)	
Trajectory 2	1008 (54.5)	143 (52.2)	
Trajectory 3	157 (8.5)	61 (22.3)	
Trajectory of diastolic blood pressure change during pregnancy			0.87
Trajectory 1	805 (43.5)	115 (42.0)	
Trajectory 2	956 (51.6)	141 (51.5)	
Trajectory 3	90 (4.9)	18 (6.6)	
Trajectory of systolic blood pressure change during pregnancy			0.16
Trajectory 1	871 (47.1)	115 (42.0)	
Trajectory 2	895 (48.4)	141 (51.5)	
Trajectory 3	85 (4.6)	18 (6.6)	
Mean (SD) maternal hemoglobin concentration at enrollment	120.3 (28.6)	121.2 (14.7)	0.61
Trajectory of hemoglobin concentration change during pregnancy			0.79
Trajectory 1	304 (16.4)	47 (17.2)	
Trajectory 2	1449 (78.3)	215 (78.5)	
Trajectory 3	98 (5.3)	12 (4.4)	

Values are numbers (percentages) unless stated otherwise

^§^Chi-square test, *t* test, and Wilcoxon rank-sum test were used as appropriate

### Maternal and early life risk factors of childhood overweight/obesity identified by machine learning

**Fig. 2:**
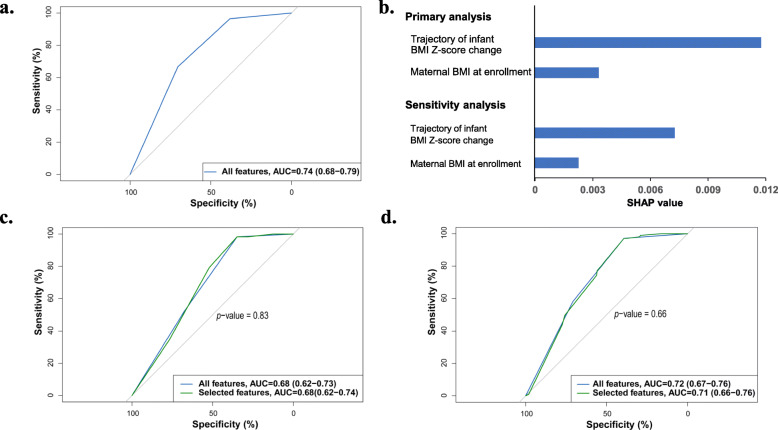
Machine learning-identified features effectively predict future childhood overweight/obesity. **a** Receiver operating characteristic curves (ROC curves) of the predictive models based on all input features in the validation cohort (*n* = 381). **b** The average impact of individual features on childhood overweight/obesity risk. We took the mean absolute value of SHAP values for the selected features to get their average impact on predicting childhood overweight/obesity. **c** Comparison of the performance of the predictive model based on all features with that based on selected features only in the test cohort (*n* = 382). **d** Comparison of the performance of the predictive model based on all features with that based on selected features only in the sensitivity analysis (childhood overweight/obesity defined according to criteria based on data derived from Chinese children)

Figure 2a showed the ROC curve with an AUC of 0.74 (95% CI 0.68 to 0.79) in the validation set, which reflected the accuracy of the prediction model with all inputted features in the model. Two most important features, trajectory of infant BMI *Z*-score change and maternal BMI at enrollment, were identified from the machine learning algorithm (Fig. 2b). Figure 2c showed the performances of the model in the test set, and the selected features showed similar predictive capacity compared with all features (AUC 0.68 vs. 0.68; *p* = 0.83, DeLong’s test).

The sensitivity analyses identified the same two features (i.e., trajectory of infant BMI *Z*-score change and maternal BMI at enrollment), and the ranking of the two features’ SHAP value was unchanged (Fig. 2b). In the independent test cohort, the AUC for childhood overweight/obesity classification using the two features was 0.71 (95% CI 0.66 to 0.76), which was comparable to that yielded based on all features (0.72, 95%, 0.67 to 0.76, Fig. 2d). Moreover, using the EFS tool, we also successfully replicated our results, which consistently showed the trajectory of infant BMI *Z*-score change during the first year of corrected age and maternal BMI at enrollment were the top two important features depending on the ensemble importance (Supplemental Figure 2a and b).

Participants belonging to trajectory 2 or 3 of BMI *Z*-score change have a positive SHAP value for this feature, while others belonging to trajectory 1 have a negative SHAP value (Supplemental Figure 3a). Therefore, we defined trajectories 2 and 3 as unfavored patterns of BMI *Z*-score change, while trajectory 1 was defined as a favored pattern. Similarly, positive SHAP values were assigned to maternal BMI at enrollment if it was > 20.8 kg/m^2^ (Supplemental Figure 3b).

### Association of early life feeding practice with trajectories of BMI *Z*-score change among preterm infants

**Fig. 3:**
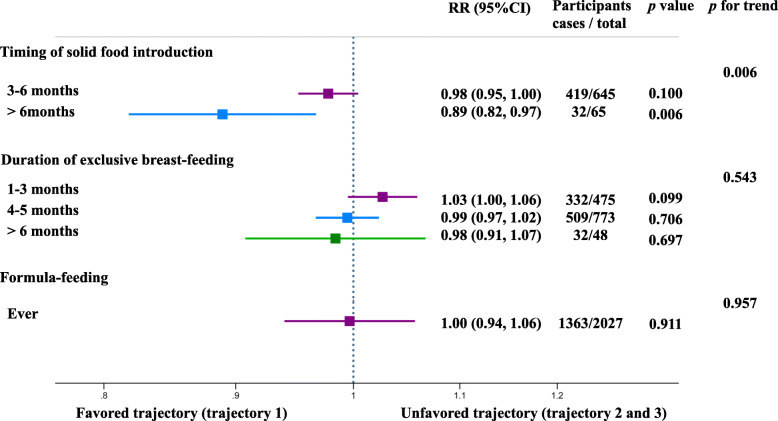
Association of the feeding practices with trajectory of BMI *Z*-score change early in life. Trajectory 2 and trajectory 3 were combined as an unfavored trajectory. Poisson regression was used to estimate the risk ratio (RR) and 95% confidence interval (CI) of unfavored trajectories, adjusted for mode of delivery, age at birth of offspring, maternal education status, occupation, parity, maternal BMI at enrollment, maternal smoking status, maternal drinking status, and newborn birth weight. For the three modifiable feeding practices, the reference group was ≤ 3 months, < 1 month, and never, respectively

When combining the trajectories 2 and 3 as an unfavored pattern of BMI *Z*-score change, our results showed that introducing solid foods after 6 months of corrected age was associated with a 11% lower risk (RR, 0.89; 95% CI, 0.82 to 0.97) of being in the unfavored trajectory of BMI *Z*-score change, compared with early introduction (≤ 3 months of corrected age, Fig. 3). When treating trajectories 2 and 3 separately, the RR of unfavored trajectory was 0.89 (95% CI, 0.81 to 0.97) and 0.79 (95% CI, 0.65 to 0.96), respectively (Supplemental Figure 4). We did not observe a significant association between the duration of exclusively breast-feeding and the risk of being in the unfavored trajectory (Fig. 3).

The mediation analysis confirmed that there was a significant indirect effect of timing of introducing solid foods via trajectory of BMI *Z*-score change early in life on the future childhood BMI *Z*-scores after adjusting for potential confounders (beta = − 0.09, 95% CI, − 0.17 to − 0.01, *p* < 0.05). The effect ratio indicated that the trajectory of BMI *Z*-score change early in life explained 80% of the total effect of timing of introducing solid foods on childhood BMI *Z*-scores (Table 2).

**Table 2:** Mediation of the associations between timing of solid foods introduction and childhood BMI *Z*-score by trajectory of BMI *Z*-score change during the first year of corrected age

	Coefficient	Standard error	***p*** values	Bootstrap 95% CI	Effect ratio
Total effect	− 0.12	− 0.05	0.01	–	–
Direct effect	− 0.02	0.02	0.31	–	–
Indirect effect	− 0.09	0.04	0.02	(− 0.17, − 0.01)	0.80

Analyses are adjusted for mode of delivery, gestational age, age at birth of offspring, maternal education status, occupation, parity, maternal BMI at enrollment, maternal smoking status, maternal drinking status, and newborn birth weight

## Discussion

Our findings suggest that 12.9% of the prematurely born infants progress to overweight or obesity at age 4–7 years. The trajectory of BMI *Z*-score change during the first year of corrected age is the most important predictor for childhood overweight/obesity, and introducing solid foods after 6 months of corrected age is a recommended feeding practice that could potentially lower the risk of unfavored trajectories of BMI *Z*-score change.

Accumulating evidence has demonstrated an increasing prevalence of overweight and obesity among preterm born children over the past decades [23, 24]. The accumulated proportion of children with childhood overweight/obesity was 12.9% in the present study, which was higher than the overweight/obesity rate for term peers in the same cohort at ages 4–7 years [25]. Therefore, premature birth might not only lead to well-known short-term morbidities but also to later overweight/obesity. A recent study by Nicole and colleagues suggested that birth weight played critical roles in later weight gain and reported a U-shaped relationship between birth weight and future obesity [26]. Therefore, low birth weight may partially explain the high risk of childhood overweight/obesity for preterm infants. However, it remains inconsistent among prior studies about the early life risk factors of the childhood overweight/obesity among preterm children [4, 5, 23, 26, 27].

To the best of our knowledge, previous studies on this topic exclusively used a linear or logistic regression to evaluate the relationships between risk factors of interest and later overweight/obesity based on a priori assumption among preterm born children [4, 5, 26]. The LightGBM model used in the present study took advantages of artificial intelligence and learned the relationship between all collected features and outcomes without any assumption [9]. The risk factors for childhood overweight/obesity that had been widely identified using traditional statistical analyses were high birth weight, rapid postnatal weight gain, and pre-pregnancy maternal BMI [4, 5, 23, 27]. In the present study, we identified the trajectory of BMI *Z*-score during the first year of corrected age as a leading predictor. Treating the infant BMI *Z*-score as a trajectory over time could enable a more comprehensive understanding of infant BMI measures (e.g., birth weight, birth length, postnatal weight gain and its velocity) and how they may jointly influence the development of child obesity. Additionally, although the information on pre-pregnancy BMI was not available, the maternal BMI at enrollment was included in our model and was identified as an important predictor. Notably, the median gestation weeks at enrollment in the present study is 10 weeks (IQR 8.3 to 12.3), at which time point the BMI is generally similar to that before pregnancy. Collectively, these results suggest the model construction and interpretation are reliable.

Interestingly, the trajectory of BMI *Z*-score is the only one feature identified in the present study that could be potentially modified by feeding practices, such as the timing of introducing solid foods. The encouragement of breast-feeding has been a universal agreement across guidelines and recommendations; however, the timing to introduce solid foods for preterm infants is under debate. For term infants, both the WHO and American Academy of Pediatrics (AAP) recommended exclusive breast-feeding for the first 6 months, while the European Society for Pediatric Gastroenterology, Hepatology and Nutrition (ESPGHAN) recommended the introduction of complementary foods be started until at least 17 weeks of age, but no later than 26 weeks [28–32]. Moreover, direct translation of these recommendations into preterm guidelines is challenging. In contrast, the present study with a large sample size and a long follow-up period demonstrated that preterm infants may benefit from delayed introduction of solid foods to 6 completed months corrected age or later.

The main strength of this study is that we apply a machine learning algorithm to identify risk factors contributing to childhood overweight or obesity based on a large longitudinal study. This algorithm takes advantages of artificial intelligence to process complex, high-dimensional, and heterogeneous features and addresses the relationships between all collected features and outcomes without any assumption. Furthermore, a novel unified framework, SHAP, is used to interpret predictions and the identified predictive factors are robust. Additionally, we have identified the best timing of solid food introduction that may be informative for initiating early intervention to prevent childhood overweight/obesity among preterm infants.

The study has several limitations. First, many preterm children are excluded from the primary analyses due to missing follow-up data. Nevertheless, mother-child pairs included in the primary analysis and those excluded are balanced with respect to most characteristics. Second, our study is based on data from only one study in a developing country, and approximately 99% of the included infants were born at 32–36 weeks of gestation; therefore, caution should be taken in extrapolating the findings to other populations.

## Conclusions

In summary, with a novel interpretable machine learning algorithm, we find that the pattern of BMI *Z*-score change during the first year is the most important predictor for childhood obesity. Introducing solid foods at 6 months corrected age or later is a recommended feeding practice for preterm infants to mitigate the risk of unfavored pattern of BMI *Z*-score change early in life. Our results provide important public health message for preterm children that early life growth trajectory is an important target for the prevention of future overweight/obesity. Beyond feeding practice, future research could further examine the association of other maternal and infant factors which could regulate the growth trajectory of the preterm infants.

## Supplementary information

**Additional file 1.**

## Data Availability

Data of the present research is available from the corresponding author on reasonable request.

## Abbreviations

AUC: Area under the curve
BMI: Body mass index
EFB: Exclusive Feature Bundling
EFS: Ensemble feature selection
GBM: Gradient boosting machines
GOSS: Gradient-based One-Side Sampling
ROC: Receiver operating characteristic
SHAP: Shapley Additive exPlanations
