# Supplementary material for: Integration of an interpretable machine learning algorithm to identify early life risk factors of childhood obesity among preterm infants: a prospective birth cohort
Source: BMC Med. 2020 Jul 10;18:184. doi: 10.1186/s12916-020-01642-6 (PMC7350615; doi:10.1186/s12916-020-01642-6)

**Supplemental Tables and Figures**

**Supplemental Table 1 The list of summarized features.**

**Supplemental Table 2 Comparison of characteristics between included and excluded participants.**

**Supplemental Figure 1 Distribution of the childhood overweight/obesity cases (n=274) according to age.**

**Supplemental Figure 2 The rank of candidate features depending on the ensemble importance quantified by the EFS**

**(a)** The childhood overweight/obesity was defined according to the 2006 WHO Child Growth Standards (for children between 2 and 3 years old) and 2007 WHO Child Growth standards (for children older than 5 years). **(b)** The childhood overweight/obesity was defined according to criteria which was used to screen overweight and obesity in Chinese children.

**Supplemental Figure 3 The marginal effect of individual selected features on the prediction of childhood overweight/obesity**

We plot the SHAP values of every feature for every sample, and SHAP value greater than zero indicates that the feature may increase the risk of childhood overweight/obesity, otherwise, decrease the risk. **(a)** plotted SHAP value for the feature of trajectory of infant BMI Z-score change during the first year of corrected age; **(b)** plotted SHAP value for the feature of maternal BMI at enrolment; red dash line indicates the cut-off point of the maternal BMI that corresponds to a different direction of prediction.

**Supplemental Figure 4 Sensitivity analysis for the association of modifiable feeding practices with trajectory of BMI Z-score change early in life.** Trajectory 2 and trajectory 3 were treated as a unfavored trajectory separately. Poisson regression was used to estimate the risk ratio (RR) and 95% confidence interval (CI) of unfavored trajectories, adjusted for mode of delivery, age at birth of offspring, maternal education status, occupation, parity, maternal BMI at enrollment, maternal smoking status, maternal drinking status and newborn birth weight. For the three modifiable feeding practices, the reference group was ≤3 months, <1 month and never, respectively.

| **Features** |
| --- |
| Sex |
| Gestational weeks |
| Fetal heart rate at the last assessment |
| Delivery mode |
| Parity |
| Birth weight |
| Birth length |
| Apgar score at 1 min |
| Apgar score at 5 min |
| Duration of breastfeeding |
| Use of formula |
| Timing of complementary foods introduction |
| Maternal age at pregnancy |
| Maternal age of menarche |
| Maternal education |
| Maternal occupation |
| Maternal BMI at enrollment |
| Maternal hemoglobin concentration at enrollment |
| Smoking during pregnancy |
| Drinking during pregnancy |
| Trajectory of BMI Z-score change during the first year of life |
| Trajectory of BMI change during pregnancy |
| Trajectory of diastolic blood pressure change during pregnancy |
| Trajectory of systolic blood pressure change during pregnancy |
| Trajectory of hemoglobin concentration change during pregnancy |

**Supplemental Table 1 The list of summarized features**

| **Characteristics**§ | **Included** |  | **Excluded** | | |
| --- | --- | --- | --- | --- | --- |
|  | **n=2125** |  | **n=6144^†^** | **No. of missing value** | |
| Boys | 1262 (59.4) |  | 3484 (56.7) | | 0 |
| Overweight/obesity | 274 (12.9) |  | 207 (15.7) | | 4826 |
| Mean (SD) gestational weeks | 35.1 (1.3) |  | 34.8 (1.8) | | 0 |
| Mean (SD) fetal heart rate at the last assessment | 140.9 (6.3) |  | 140.6 (6.6) | | 275 |
| Caesarean delivery | 1268 (59.7) |  | 3361 (54.7) | | 0 |
| Mean (SD) Birth weight | 2.8 (0.52) |  | 2.7 (0.8) | | 0 |
| Mean (SD) Birth length | 48.3 (2.4) |  | 47.9 (3.0) | | 0.02 |
| Mean (SD) Apgar score at 1 min | 8.6 (1.5) |  | 8.5 (1.5) | | 72 |
| Mean (SD) Apgar score at 5 min | 9.3 (1.1) |  | 9.1 (1.2) | | 72 |
| Duration of breastfeeding |  |  |  | | 2189 |
| <1 month | 829 (39.0) |  | 2050 (51.8) | |  |
| 1-3 months | 475 (22.4) |  | 693 (17.5) | |  |
| 4-5 months | 773 (36.4) |  | 1104 (27.9) | |  |
| >6 months | 48 (2.3) |  | 108 (2.7) | |  |
| Timing of solid foods introduction |  |  |  | | 2402 |
| ≤ 3 months | 1415 (66.7) |  | 2720 (72.7) | |  |
| 4-6 months | 645 (30.4) |  | 940 (25.1) | |  |
| > 6 months | 65 (3.0) |  | 82 (2.2) | |  |
| Mean (SD) maternal age at pregnancy | 25.2 (4.1) |  | 25.9 (4.7) | | 2 |
| Mean (SD) maternal age of menarche | 14.7 (1.3) |  | 14.6 (1.3) | | 1 |
| Maternal education |  |  |  | | 10 |
| < High school | 1548 (72.8) |  | 3838 (62.6) | |  |
| High school | 380 (17.9) |  | 1023 (16.7) | |  |
| > High school | 197 (9.3) |  | 1273 (20.8) | |  |
| Maternal occupation |  |  |  | | 26 |
| Farm work/housework | 1395 (65.6) |  | 3664 (59.9) | |  |
| Routine job | 372 (17.5) |  | 1123 (18.4) | |  |
| Temporary work | 162 (7.6) |  | 348 (5.7) | |  |
| Others | 196 (9.2) |  | 983 (16.1) | |  |
| Mean (SD) maternal BMI at enrollment | 21.0 (2.9) |  | 21. 5 (3.4) | | 108 |
| Mean (SD) maternal hemoglobin concentration at enrollment | 120.4 (27.2) |  | 120.4 (20.7) | | 911 |
| Smoking during pregnancy | 2 (0.1) |  | 14 (0.2) | | 46 |
| Drinking during pregnancy | 4 (0.2) |  | 47 (0.8) | | 48 |
| *Values are numbers (percentages) unless stated otherwise  §For each characteristic, participants with missing value were excluded from the statistical summary.  †Total number of excluded participants | | | | | |

**Supplemental Table 2 Comparison of characteristics between included and excluded participants ***

**Supplemental Figure 1 Distribution of the childhood overweight/obesity cases (n=274) according to age.**


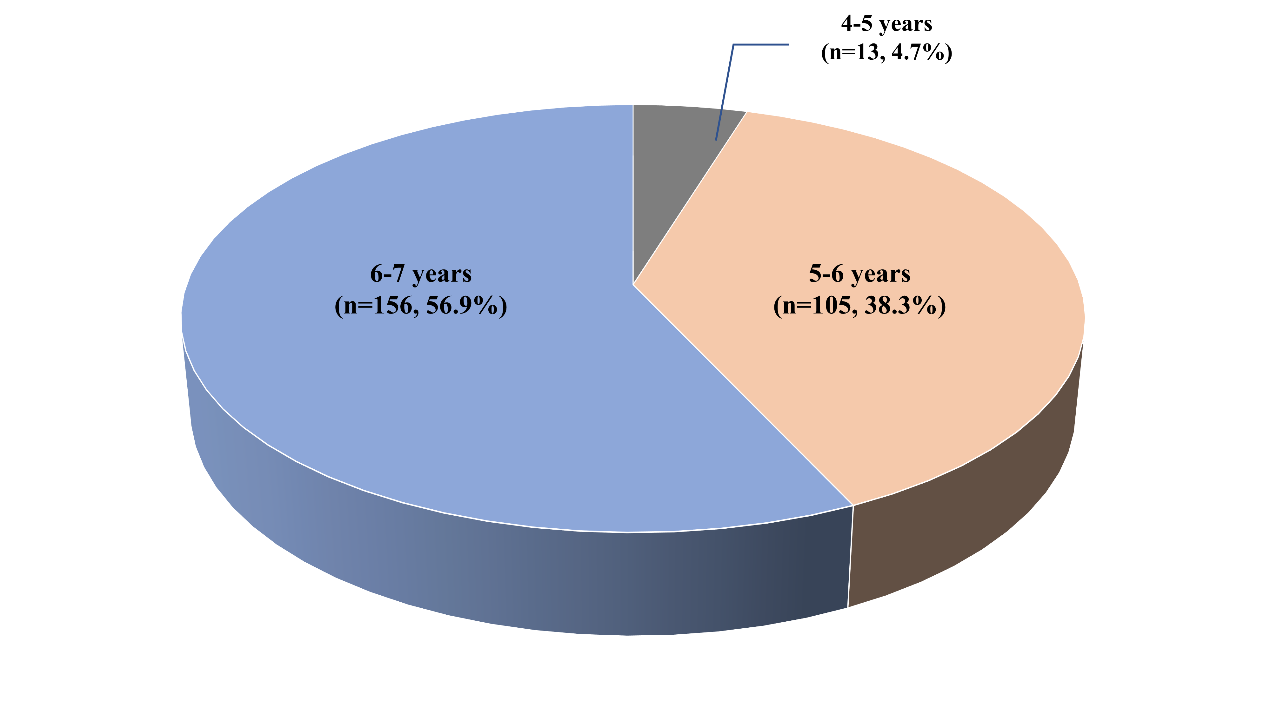


**Supplemental Figure 2 The rank of candidate features depending on the ensemble importance quantified by the EFS**

**a**


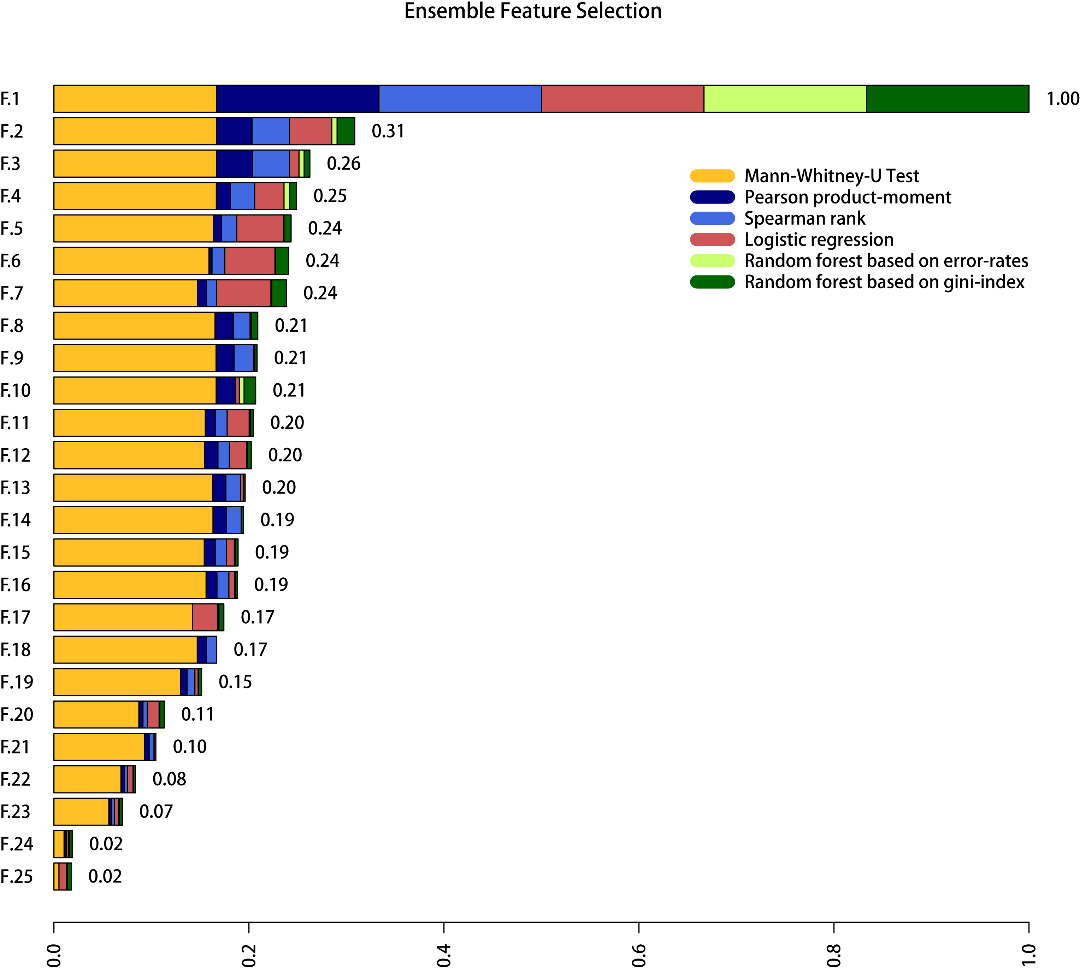


**b.**


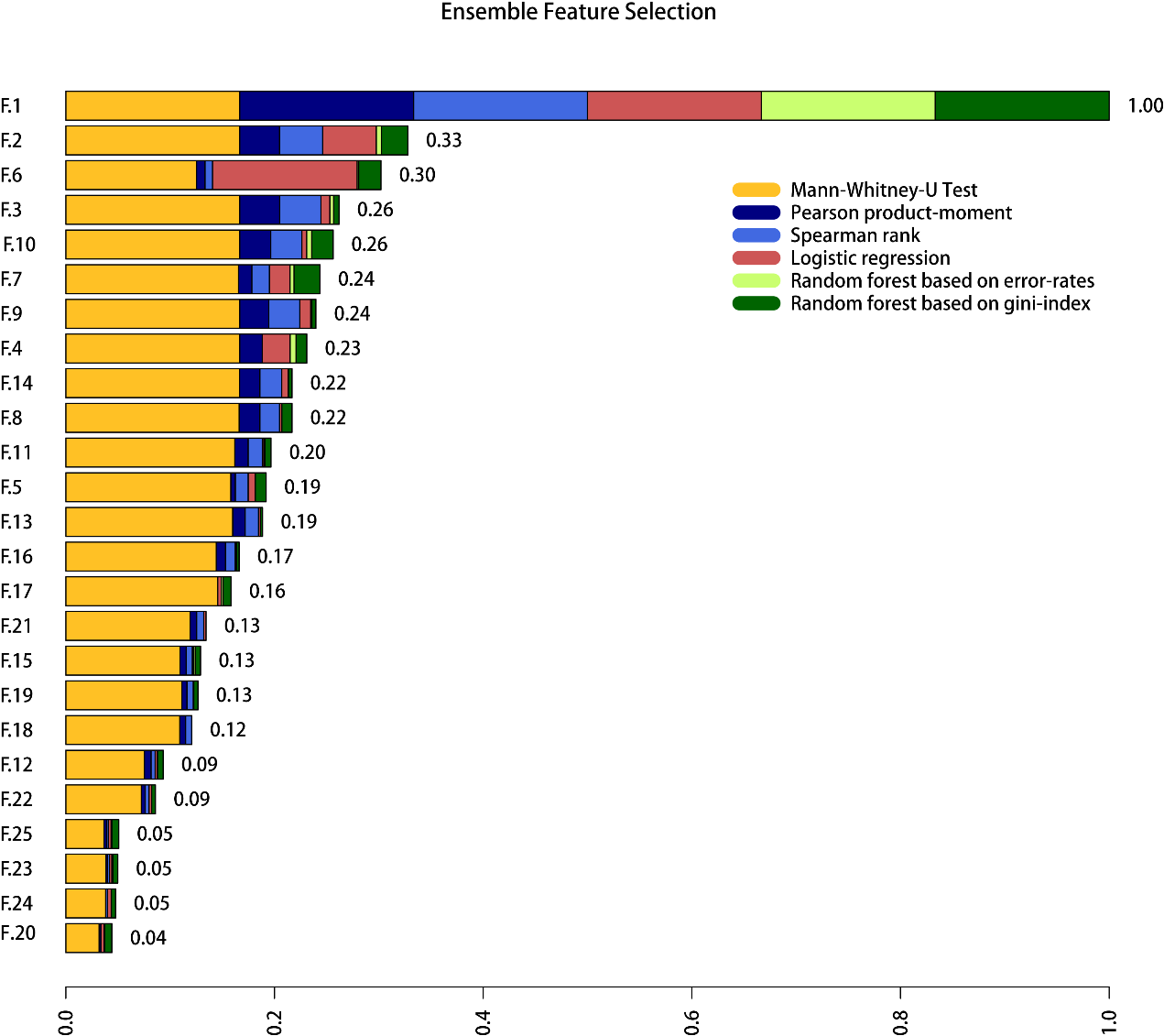


F.1: Trajectory of BMI Z-score change during the first year of life; F.2: Maternal BMI at enrollment; F.3: Trajectory of BMI change during pregnancy; F.4: Birth length; F.5: Fetal heart rate at the last assessment; F.6: Maternal hemoglobin concentration at enrollment; F.7: Maternal age at pregnancy; F.8: Maternal age of menarche; F.9: Sex; F.10: Birth weight; F.11: Apgar score at 5 min; F.12: Maternal occupation; F.13: Use of formula; F.14: Delivery mode; F.15: Trajectory of systolic blood pressure change during pregnancy; F.16: Parity; F.17: Apgar score at 1 min; F.18: Smoking during pregnancy; F.19: Maternal education; F.20: Duration of breastfeeding; F.21:Drinking during pregnancy; F.22: Trajectory of hemoglobin concentration change during pregnancy; F.23: Trajectory of diastolic blood pressure change during pregnancy; F.24: Timing of complementary foods introduction; F.25: Gestational weeks.

**Supplemental Figure 3 The marginal effect of individual selected features on the prediction of childhood overweight/obesity.**


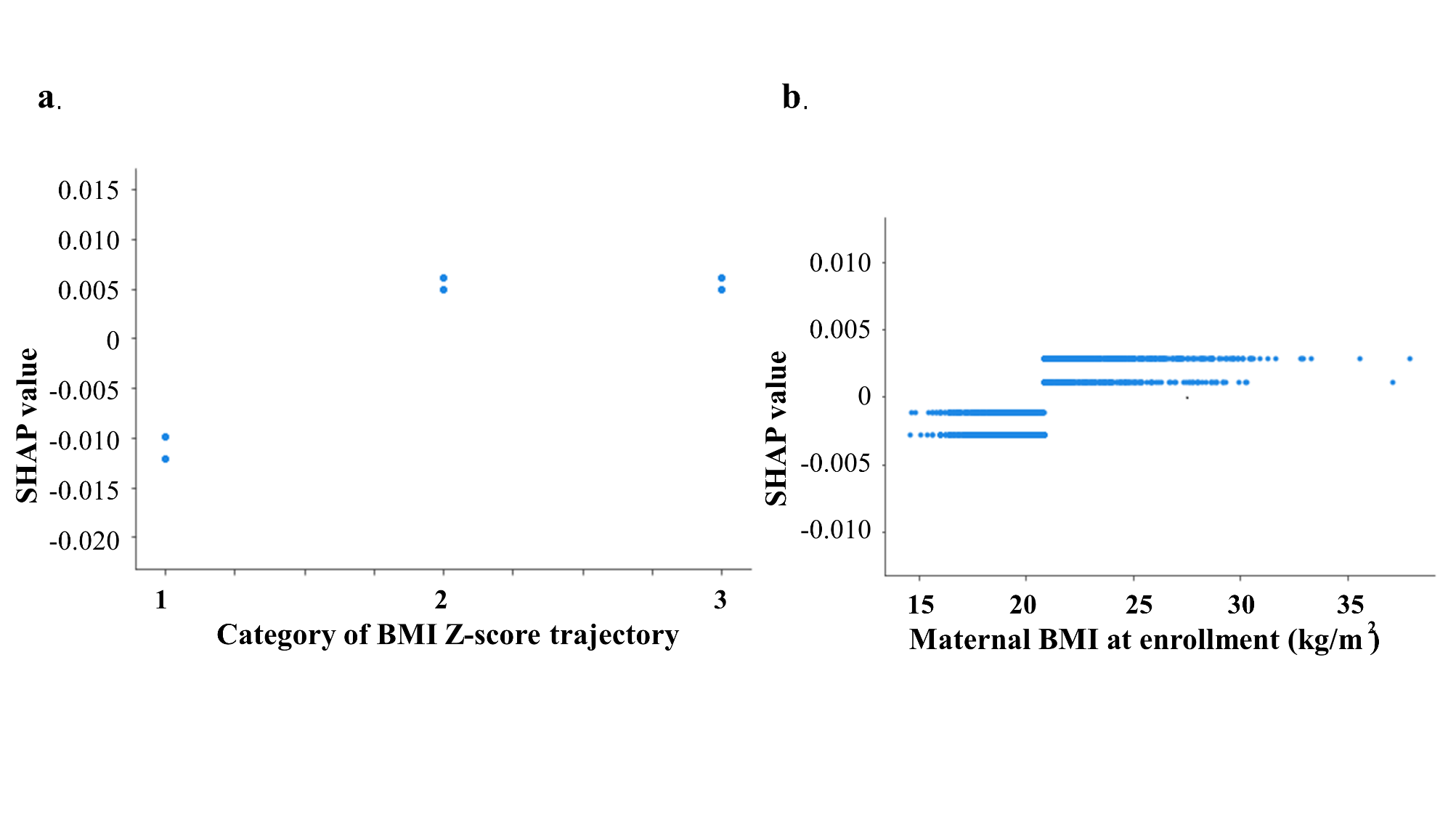


**Supplemental Figure 4 Sensitivity analysis for the association of feeding practices with trajectory of BMI Z-score change early in life**


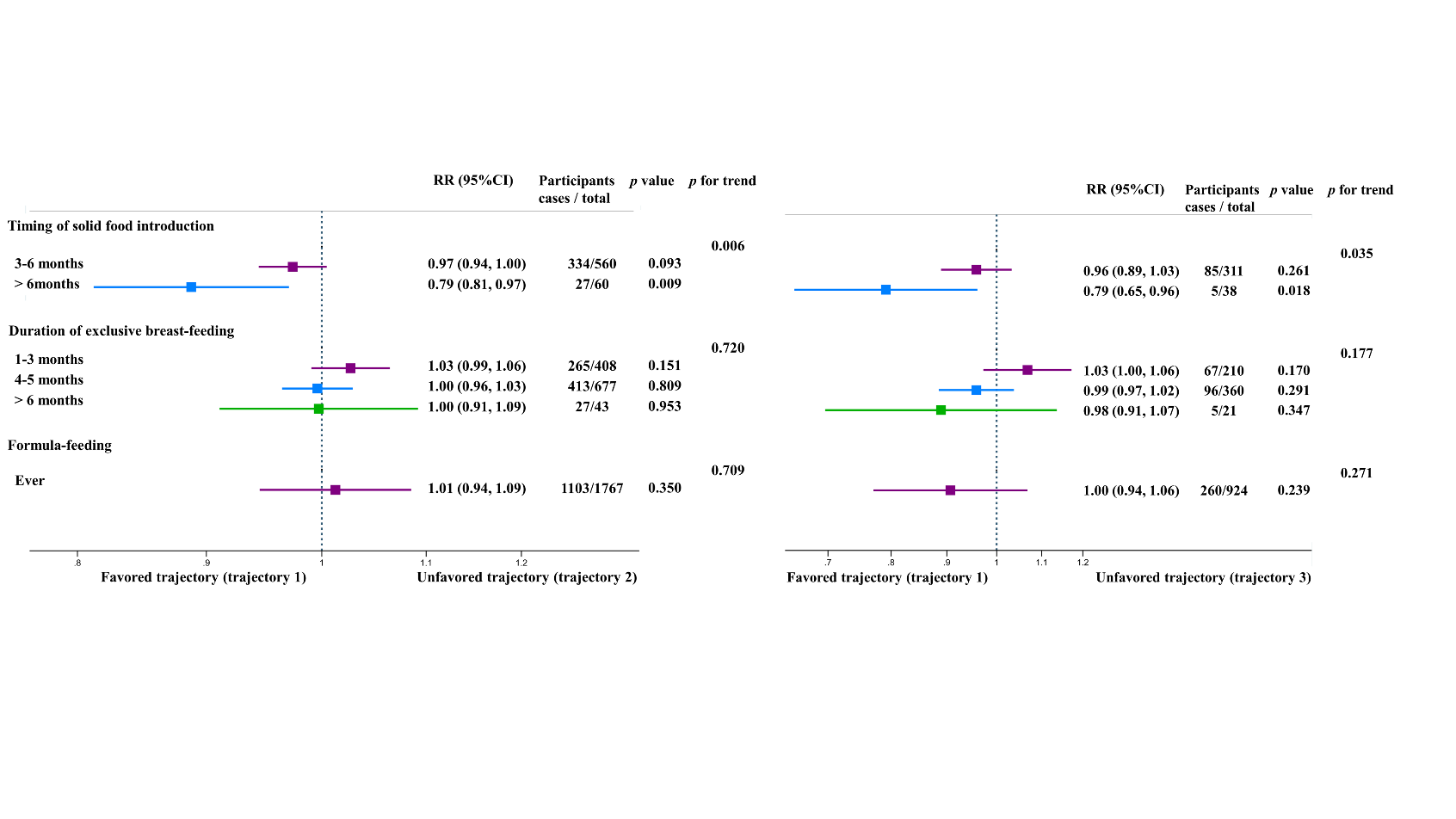

Supplement: Supplementary file 1 — Additional file 1. [file 12916_2020_1642_MOESM1_ESM.docx]
